# Integrating Plantain (*Plantago lanceolata* L.) and Italian Ryegrass (*Lolium multiflorum* Lam.) into New Zealand Grazing Dairy System: The Effect on Farm Productivity, Profitability, and Nitrogen Losses

**DOI:** 10.3390/ani11020376

**Published:** 2021-02-02

**Authors:** Omar Al-Marashdeh, Keith Cameron, Simon Hodge, Pablo Gregorini, Grant Edwards

**Affiliations:** 1 Faculty of Agriculture and Life Sciences, Lincoln University, Lincoln 7647, Canterbury, New Zealand; keith.cameron@lincoln.ac.nz (K.C.); simon.hodge@ucd.ie (S.H.); Pablo.Gregorini@lincoln.ac.nz (P.G.); Grant.Edwards@lincoln.ac.nz (G.E.); 2 School of Agriculture and Food Science, University College Dublin, Belfield, Dublin 4, Ireland

**Keywords:** farm productivity, farm profitability, grazing dairy system, Italian ryegrass, nitrate leaching, plantain

## Abstract

**Simple Summary:**

Short-term studies have suggested plantain and Italian ryegrass as alternative forages to ryegrass–white clover sward to reduce the environmental footprint of grazing dairy farms. However, the adoption of such forages by farmers will likely be limited until more certainty around its effect on farm productivity and profitability. The objective of this study was to provide multiple-year and farm-scale evidence to farmers with respect to the effects of integrating plantain and Italian ryegrass into the ryegrass and white clover-based dairy system on farm productivity, profitability, and nitrate leaching losses. Under similar farming system input, the plantain plus Italian ryegrass-based dairy system could be used by dairy farmers to mitigate the environmental footprint while maintaining farm productivity and profitability.

**Abstract:**

A two-year farm system study was conducted at Canterbury, New Zealand to evaluate the effects on farm productivity, profitability, and nitrogen (N) losses of integrating plantain (*Plantago lanceolate* L.) and Italian ryegrass (*Lolium multiflorum* Lam.) into a ryegrass and white clover (RGWC)-based dairy system. Three farm systems were compared: (1) a lower input RGWC-based system (LIRG) with stocking rate of 3.5 cow/ha, annual N fertiliser rate of 150 kg/ha, and imported feed level of <1.2 t DM/cow/year; (2) a lower input ryegrass + plantain-based system (LIRG + PL) with a stocking rate of 3.5 cow/ha, annual N fertiliser rate of 150 kg/ha, and imported feed level of <1.2 t DM/cow/year; and (3) a higher input RGWC-based system (HIRG) with a stocking rate of 5.0 cow/ha, annual N fertiliser rate of 300 kg/ha, and imported feed level of >1.2 t DM/cow/year. Cows in the LIRG + PL system grazed a diverse mix of Italian ryegrass, perennial ryegrass, white clover, and plantain (60% of farmlet area), and a mixed sward of plantain–white clover (40% of farmlet area). The average annual herbage harvested was similar between LIRG + PL and LIRG (11.7 t DM/ha), but greater in HIRG (12.7 t DM/ha) with the increased N fertiliser rate. During the calving to dry-off period, the average imported supplement feed per ha was higher in HIRG (8.0 t DM) compared with LIRG (3.2 t DM) and LIRG + PL (3.7 t DM). Average milk solid production (MS; fat + protein) was similar in LIRG + PL (1640 kg/ha) and LIRG (1622 kg/ha), but greater in HIRG (2130 kg/ha). Estimated profitability (NZD/ha) at milk price of NZD 6.5/kg MS was 10% greater for HIRG than LIRG + PL and LIRG, and similar (<1.5% numerical difference) between LIRG + PL and LIRG. The average estimated annual N leaching loss from the LIRG and LIRG + PL was 31% and 56% less than the loss from the HIRG. These large reductions in N leaching loss were achieved without a large decrease in profitability (i.e., LIRG and LIRG + PL compared to HIRG). In addition, the estimated reduction in N losses from the LIRG + PL system compared to LIRG suggests that an Italian ryegrass + plantain-based dairy system is a viable strategy to reduce the environmental footprint while maintaining farm profitability. However, the environmental benefits of plantain and Italian ryegrass estimated in this study require further confirmation through direct measurements at full farm level.

## 1. Introduction

Pastoral livestock production systems are popular in areas with temperate climates because of their consistent supply of high-quality feed, low operational cost, and simplicity of establishment and management [1,2]. The New Zealand pastoral dairy system is primarily based on swards comprising perennial ryegrass (*Lolium perenne* L.) and white clover (*Trifolium repens* L.). However, urine patches from cows grazing such swards are a major contributors to on-farm nitrogen (N) pollution. This is caused by the ryegrass–white clover (RGWC) sward delivering N in excess of animal nutritional requirements [3,4,5]. Grazing dairy cows excrete more than 50% of their N intake in their urine [6,7], which produces highly concentrated N patches at rates equivalent to application of 653–1366 kg N/ha [4]. This high N load per urine patch exceeds the capability of plant N uptake, resulting in excess N being lost through nitrate leaching and nitrous oxide emission [3,4,8].

Since the implementation of nitrate leaching regulations [9], New Zealand dairy farmers have been under pressure to reduce their environmental footprint while maintaining farm profitability. Several mitigation strategies have been explored to reduce overall N leaching, both at the animal level in terms of urine volume and N concentration [6,10,11,12] and at the soil level in terms of preventing run-off and N capture [8,13,14]. These mitigation strategies have focused on different pathways and include: (1) reducing animal N intake or altering N partitioning (i.e., more N towards milk and faeces and less to urine) by dietary manipulation (e.g., feeding high-sugar grass to increase energy to N ratio in the diet) [6,11,15]; (2) breeding cattle which produce urine with a lower N concentration [16,17]; and (3) increasing N uptake from soil by establishing swards comprising plant species varying in functional traits, such as growth activity during the season (e.g., Italian ryegrass *Lolium multiflorum* Lam. with greater growth at low temperatures) [13,14]. However, the benefits of these strategies in mitigating the environmental footprint of dairy systems were investigated in a short time frame and/or over a small physical scale, such as in lysimeter studies [18]. Whether the suggested mitigation strategies function over larger physical and temporal scales, or whether there are secondary effects on farm productivity and profitability, remains unclear.

Plantain (*Plantago lanceolate* L.) has been shown to produce a diuretic effect in dairy cows and heifers, resulting in increased volume and reduced N concentration of urine [10,19,20,21]. Furthermore, secondary compounds in plantain alter N partitioning in the cow, resulting in more N in the milk and faeces and less in the urine [19]. Lysimeter studies have shown a reduction in nitrate leaching from cows grazing an Italian ryegrass, plantain, and white clover mix sward compared with leaching from a conventional RGWC sward [18]. This reduction in nitrate leaching was the result of lower urinary N concentration from cows grazing the plantain in addition to higher N uptake by the Italian ryegrass. However, the environmental benefits of such a mixed sward appear less convincing at farm level, and the voluntary uptake of these swards by farmers will likely be limited until there is more certainty around its effect on farm productivity and profitability.

The overall aim of this study was to provide evidence to farmers with respect to the effects of integrating plantain and Italian ryegrass into the RGWC-based dairy system on farm productivity, profitability, and nitrate leaching losses. Therefore, the practical objectives of the current study were to evaluate, in a two-year farm system context, the impacts of integrating plantain and Italian ryegrass into the dairy system on farm production, economic efficiency, and N losses when compared with two common RGWC-based systems differing in their levels of imported feeds and N fertiliser input.

## 2. Materials and Methods

The study was conducted over two lactation seasons from August 2017 to May 2019 at the Lincoln University’s Ashley Dene Research and Development Station (ADRDS), Springston, Canterbury, New Zealand (−43.65° N, 172.33° E), on a free-draining Balmoral/Lismore stony silt loam soil. During the period of study, cumulative rainfall was 681 mm in 2017/18 and 639 mm in 2018/19 milking year, and average air temperature was 12.3 °C in 2017/18 and 12.2 °C in 2018/19. The study was carried out with the approval of the Lincoln University Animal Ethics Committee (AEC 2017-12).

### 2.1. Experimental Design

In August 2017, a total of 271 New Zealand Holstein–Friesian dairy cows were randomly allocated to three unreplicated experimental farmlets: (1) lower input RGWC-based system (LIRG) with lower stocking rate (SR; 3.5 cow/ha) and herbage received 150 kg N/ha per year; (2) lower input ryegrass plus plantain-based system (LIRG + PL) with lower SR (3.5 cow/ha) and herbage received 150 kg N/ha per year; and (3) higher input RGWC-based system (HIRG) with higher SR (5.0 cows per ha) and herbage received 300 kg N/ha per year.

A total of 67.5 ha of 2.25-ha paddocks were allocated to the farmlets (10 paddocks per farmlet). The experimental paddocks were in one block and were evaluated to ensure that they were balanced among the three farmlets for topography and distance to the milking parlour. All farmlets were 22.5 ha in area, with either 113 (28 primiparous and 85 multiparous) cows in the HIRG farmlet or 79 (22 primiparous and 57 multiparous) cows in each of the LIRG and LIRG + PL farmlets. The animals were randomly allocated to the farmlets; however, herds were balanced for age (3.7 ± 1.52 year; mean ± SD), live weight (LW; 496 ± 70.7 kg; before calving), calving date (for multiparous cows; 12 August ± 8.9 days), breeding worth index (BW; a measure of genetic merit accounting for economic values for fertility, production, residual survivor, LW, and body condition score (BCS); 95 ± 28.8), and production worth (PW; a measure of genetic merit accounting for economic values for milk volume, milk fat and protein, and LW; 108 ± 68.9).

### 2.2. Experiment Management

#### 2.2.1. Herbage Establishment

The RGWC paddocks were sown during autumn in 2016 (16 paddocks; total area of 36 ha) and 2017 (4 paddocks; total area of 9.0 ha) with a seed mixture of perennial ryegrass (*Lolium perenne* L.; cv. Base/One50 20 kg/ha) and white clover (*Trifolium repens* L. cv. Legacy; 5 kg/ha). Paddocks with different sowing dates were allocated to the two ryegrass-based farmlets (HIRG and LIRG) to ensure that these farmlets were balanced for herbage establishment date. The LIRG + PL herbage area comprised 13.5 ha of diverse mix and 9.0 ha of plantain–white clover mix sward. During autumn 2016, the diverse sward was sown with a seed mixture of perennial ryegrass (cv. Base/One50 12 kg/ha), Italian ryegrass (*Lolium multiflorum* Lam. cv. Asset; 6 kg/ha), plantain (*Plantago lanceolata* L. cv. Tonic; 4 kg/ha), and white clover (cv. Legacy; 5 kg/ha), and it was topped up in the following autumn (via direct drilling) with Italian ryegrass (10 kg/ha) and plantain (4 kg/ha). The plantain–white clover mix sward was sown during autumn in 2017 with a seed mixture of plantain (cv. Tonic 14 kg/ha) and white clover (cv. Legacy; 4 kg/ha).

#### 2.2.2. Nitrogen Fertiliser and Irrigation

Nitrogen was applied as urea fertiliser in 9 to 10 applications to the farmlets annually. The rate of N fertiliser applied on each occasion was 15 kg N/ha for the LIRG and LIRG + PL farmlets and 30 kg N/ha for the HIRG farmlet. The total annual amount of N fertiliser applied to each of the HIRG, LIRG, and LIRG + PL farmlets was 271, 153, and 156 kg per ha in 2017/18, and 287, 144, and 145 kg per ha in 2018/19, respectively. The timing of application was the same for all farmlets, being within 1–3 days after the grazing event during spring, summer, and early autumn. During the period of this study, no maintenance fertiliser was applied to any of the farmlets. Soil moisture was monitored via soil sensors (Aquaflex Soil Moisture Sensor, Streat Instruments, New Zealand) buried into the root zone at one location within the experimental site, while two central pivot irrigators were managed to maintain soil moisture content for optimal herbage growth. Generally, herbage was irrigated during the dry periods in late spring, summer, and early autumn (November to March). The full Irrigation Water Take Consent of 548 mm per year (5481 m^3^/ha) was reached during both milking years. The farm dairy effluent was applied to approximately 70% of the area in each farmlet, at an application rate of 5 mm from October to April. This effluent was applied via a separate pipeline and a set of secondary sprinklers attached under the two irrigation pivots. Across the two milking seasons, an average of 160, 138, and 146 mm effluent was applied per year on the HIRG, LIRG, and LIRG + PL farmlet areas, respectively.

#### 2.2.3. Grazing Management and Supplement Feeding

Cows were rotationally grazed through their respective farmlet area and managed similarly across the farmlets, except for variation in intergrazing intervals and the amount of supplement offered. Operational management decisions were made weekly, based on the change in feed supply (herbage growth) and demand (animal nutritional requirements) as described by Webby and Bywater [22]. The herbage cover (measured as compressed herbage height above ground) was determined weekly for each paddock using a rising plate meter (RPM, Jenquip, Fielding, New Zealand), and paddocks were sorted in a “feed wedge” (a bar graph sorting paddocks from longest to shortest herbage height) to determine rotational grazing order, as described by Holmes and Roche [23]. Cows grazed fresh herbage each day and were allocated to the paddock with the longest herbage height (average compressed pre-grazing herbage height of 7.0–8.0 cm above ground). Cows were shifted into a new herbage area when the desired post-grazing height (average compressed post-grazing height 3.5–4.0 cm above ground) was achieved, as recommended by [23].

The grazing rotation length was set to match supply and demand, although when herbage supply was in deficit (low herbage growth rate) and unable to meet the cows’ demands, a supplement was fed. On the other hand, herbage is usually conserved as silage or hay when growth rate exceeds animal demand. However, this situation did not arise in this study. The offered supplement was a 50:50 mixture of maize silage, and grass silage or lucerne silage (depending on availability), and allocated to cows after morning milking on a concrete feeding pad. Cows were able to finish their supplement (at >95% utilisation) in 1–2 h depending on quantity. However, when daily quantity provided was >6 kg dry matter (DM) per cow (1.2% of LW), supplement was fed over two meals (one meal after morning and evening milking).

#### 2.2.4. Animal Management

Animals were managed in a seasonal milk production system, with all cows dried-off in winter (June and July) and the start of calving was in early August. In both milking years, calving distribution was similar among the farmlet treatments. In 2017/18, however, cows with early calving dates were selected to start the farmlet study; more than 50% of cows calved within the first two weeks of calving (by mid-August), and the remaining cows calved by the 4th week (end of August). In 2018/19, a normal calving distribution (over 8–9 weeks) was allowed, with approximately 40% of cows calved in two weeks (by mid-August), 40% calved within the following 4 weeks (late August to early September), and the remaining cows calved in week 7 and 8 (mid to late September).

All cows were visually scanned for behavioural signs of oestrus before the start of the breeding season (late October), with paint applied to the tops of cows’ tails to identify those exhibiting mounting behaviour. Cows that did not exhibit behavioural sings of oestrus were examined by the veterinarian. Artificial insemination was performed for 10 weeks (late October to early January), and cows were diagnosed for pregnancy at six weeks after the end of the breeding season. The pregnancy rate was 88%, 91%, and 94% in 2017/18 and 81%, 84%, and 85% in 2018/19 for HIRG, LIRG, and LIRG + PL, respectively.

In May 2018, at the end of the 2017/18 milking year, 15%, 12%, and 8% of cows in HIRG, LIRG, and LIRG + PL, respectively, were replaced by primiparous cows. The culled cows were determined based on pregnancy failure, health, and age. The herds across the farmlets were evaluated again to ensure that they were balanced for age (4.3 ± 1.62), BW (99 ± 32.3), and PW (113 ± 68.7) at the start of 2018/19 milking year. Decision rules around dry-off dates were based on herbage availability and individual cow BCS. In 2017/18, all cows were dried-off on the 22nd of May and immediately shifted off the farmlet area to graze a winter crop (fodder beet; *Beta vulgaris* L.). All cows were managed similarly during the dry-off period and not returned to the farmlet area until 2–4 days before the expected calving date. In 2018/19, 10% of cows within each farmlet were dried-off on 17th of April and the remaining cows on 7th of May.

### 2.3. Measurements

All measurements were conducted during the lactation period from calving to dry-off date, while cows were grazing on the “milking platform”—the land supporting the lactating cows only. After cows were dried-off, they were shifted off the milking platform to graze on a winter crop (fodder beet), where no measurements were conducted. Cows were returned to the milking platform immediately before calving. No animals grazed on the milking platform while lactating cows were off-farm during winter. Therefore, the herbage harvested by milking cows is a true estimate of annual herbage yield of the milking platform.

Every fortnight, pre-grazing herbage samples were collected from one paddock in each farmlet (the paddock to be grazed next by cows) to determine the chemical and botanical composition. The herbage samples (approximately 15 samples per paddock) were cut with clippers above grazing height (3.5 cm above ground) along a diagonal line across the paddock. All samples were mixed and subsampled. One subsample was separated into its ryegrass (combination of perennial and Italian ryegrass for diverse sward in LIRG + PL), white clover, plantain, dead material, and weed species components and dried at 60 °C for 48 h. The dry weight of each component was recorded and the proportion of each species determined as a % of the whole sward. A second subsample was dried at 60 °C for 48 h, ground to 1 mm, and analysed for chemical composition using near-infrared spectrophotometry (Foss NIRSystems 5000, FOSS NIRSystems Inc., Laurel, MD, USA). The supplement feed was sampled at least once per month to determine the DM content and chemical composition, following the same method described for herbage. Nutritive value (mean ± SD) of supplement (maize silage, grass silage, and lucerne silage) fed to cows during 2017/18 and 2018/19 milking year is presented in Table 1. Metabolisable energy (ME) of feed was estimated based on the equation [ME (MJ/kg) = digestible organic matter content × 0.016 (g/kg of DM)] [24].

Herbage DM intake (DMI) was estimated from mean daily milk energy output plus cow maintenance energy requirement for LW change and activity using equations from Holmes, Brookes [25], where:

ME maintenance (MJ/day) = 0.60 MJ/kg LW^0.75^

ME lactation (MJ/day) = [(0.376 × milk fat% + 0.209 × milk protein%) + 0.976) × milk yield L/day]/k

ME Activity = 0.0037 MJ/kg LW per horizontal km walked

The mean daily milk energy output was measured based on the cow’s monthly test for milk composition and average daily milk yield during that month. The efficiency at which energy was used for milk production (k) was assumed to be 65% [25]. The LW gain/loss was measured for individual cows (kg/day) by calculating the difference in LW over one month. The energy required for 1 kg of LW gain was assumed to be 32 MJ/cow/day, and energy supplied by mobilising 1 kg of LW was assumed to be 25 MJ/cow/day [25,26]. Once pregnant cows were identified in February, ME requirements for pregnancy were calculated based on expected calf weight at birth (35 kg for Friesian–Jersey cross) and days since conception as shown by Holmes, Brookes [25]. On average, pregnancy requirements of 1.1, 2.1, and 4.0 MJ were added to the daily ME requirement of cows during March, April, and May, respectively. Supplement offered was weighed in a mixer wagon (Trioliet SoloMix, CLAAS harvest Centre, Christchurch, New Zealand) at each supplementation event, and ME intake from the supplement was calculated assuming 100% utilisation. Herbage DMI was then calculated using the following equation:

Herbage DMI (kg DM/day) = [(ME maintenance + ME lactation + ME activity +ME LW change + ME pregnancy) − total ME intake from supplement]/herbage ME (MJ/kg DM)

Cows were milked at 05:30 a.m. and 14:30 p.m., and the volume of individual cow milk yield was automatically recorded (Afimilk, Kibbutz Afikim, Israel) at each milking. During the period from October to May in 2017/18 and August to May in 2018/19, once per month and coinciding with feed sampling, milk composition (fat and protein) was determined on individual p.m. and a.m. milk samples (CRV Ambreed, Hamilton, New Zealand) using a Milkoscan milk analyser (Foss Elictric, Hillerød, New Zealand). Individual cow LW was recorded by an automatic walk-over weighing system (Waikato Milking Systems, Hamilton, New Zealand) after each milking, while cows were leaving the milking shed. During the period from October to May in 2017/18 and August to May in 2018/19, once per month, the BCS of cows was determined by an experienced observer, based on a 10-point scale, where 1 is emaciated and 10 is obese [27].

In 2017/18, to calculate individual cow annual MS production, milk solid production (MS; fat + protein kg/cow/day) was averaged across the measurement months and multiplied by the number of days in milk. Similarly, to calculate individual cow cumulative herbage DMI from calving to dry-off, daily herbage DMI was averaged across the measurement months and multiplied by the lactation length (days). This method included an estimation of milk production and herbage DMI for August and September, where MS concentration was not measured.

The economics of each farmlet were simulated using a commercial modelling tool, FARMAX Dairy (Version 8.0.1.19 FARMAX, Hamilton, New Zealand; http://www.farmax.co.nz/). Actual production data for each of the milking years studied were used to give economic values per farmlet. The model database for dairy farming operating costs and farm expenditure in the Canterbury area was used to estimate farm working expenses. The milk price of NZD 6.5 per kg MS used is the average of actual milk payout by Fonterra Co-operative Group Limited across the two milking years (NZD 6.69 and 6.39 per kg MS in 2017/18 and 2018/19, respectively). Farm profitability (before tax) was calculated as total revenue (from net milk and livestock sales) minus total farm working expenses. The total farm working expenses were the sum of labour/wages, livestock and feed expenses, cost incurred for grazing livestock replacements and wintering cows off farm, expenses such as fertiliser, irrigation, weed and pest control, vehicle expenses, and overhead expenses including administration, insurance, and depreciation. Supplementary feed was modelled as imported feed to the farmlet at similar purchase price of NZD 330 (2017/18) or NZD 320 (2018/19) per t DM of maize silage, Lucerne silage, or grass silage. These costs are accurate and reflect the average purchase price for the silages used in each of the studied years. For modelling purposes, the same stock replacement percentage each year (20%) was used among the farmlets.

The farm N surplus, nitrate leaching losses, and nitrous oxide emission were simulated using a scientific research-based modelling tool called Overseer^®^ (version 6.3.4; Wellington, New Zealand; https://www.overseer.org.nz/). A detailed technical description on how Overseer^®^ works was provided by [28]. The model estimates N surplus and nitrate leaching based on production input and output [29]. However, the model version used also takes into account the effects of plantain on urinary N excretion by animals and the subsequent nitrogen load. Such an effect is estimated based on studies undertaken as part of the Forages For Reduced Nitrate Leaching Programme, which assessed the impact of various forages including plantain on nitrate leaching [10,14,18,19,30,31]. The full description and sensitivity analysis for implementation of plantain in Overseer^®^ was described by [32]. Briefly, a linear effect of plantain content in diet (%Plantain; between 0% and 60%) on excretal N partitioned to urine and urine patch N load was estimated at block level as follows:

Urine proportion of excreta N = Overseer value for ryegrass × urine proportion of excretal N factor (UPF)

where UPF = 1 − ((%Plantain/60) × 0.2).

Urine patch N load = 750 × urine patch factor

where 750 kg N/ha is the N loading for a standard urine patch of cow grazing RGWC, and urine patch factor = 1 − ((%Plantain/60) × 0.4).

The used model version limited the plantain proportion in the diet to 60%, and no effect on urine N load is detected for proportions greater than 60%. The model does not take into account the effect of Italian ryegrass on nitrate leaching.

### 2.4. Statistical Analysis

All statistical analyses were performed using Genstat v19 statistical software (VSN International Ltd., Hemel Hempstead, UK). Because the three management systems were not replicated, the statistical analysis was centred on comparing the performance of the three farmlets. In general, as repeated measurements or samples were taken from the same animals or from the same paddocks within each farmlet, a mixed-model repeated-measures approach was required. In Genstat, these analyses were carried out using the restricted maximum likelihood modelling function (REML), which can account for the non-independence of measurements, repeated measurements, and provide estimated treatment means and least significant differences for pairwise comparisons of treatments. Depending on the response variable of interest, the experimental units were defined as either individual animals and/or individual paddocks within each farmlet. The data from the two study years were analysed separately.

For variables describing the composition of the diet (DM%, OM%, ADF%, NDF%, CP%, ME), the fixed factors of the model consisted of treatment, season, and the interaction term, along with paddock that the samples were collected from as a random factor (see Table 2). For the response variables related to milk production per cow (yield, MS, Fat%, Protein%), the mean values for each cow over each month were used as the response variables, and the fitted models included treatment and month as fixed explanatory factors, along with the interaction term, and individual cows included as a random factor (Table 4). This same model structure was also used for variables that described feed intake per cow, such as herbage intake and total DMI (Figure 2 and Table 3). For LW and BCS, the model included treatment and stage of lactation, and the interaction term, as fixed factors, and individual cows as a random factor (Table 4).

## 3. Results

### 3.1. Herbage Chemical Composition

The mean nutritive value of herbage grazed by cows during spring, summer, and autumn in 2017/18 and 2018/19 milking years is presented in Table 2. In 2017/18, the average herbage content of organic matter (OM; *p* < 0.001), DM (*p* < 0.001), acid detergent fibre (ADF; *p* = 0.008), and neutral detergent fibre (NDF; *p* < 0.001) were lower in LIRG + PL than LIRG and HIRG. Similarly, in 2018/19, the herbage content of OM (*p* < 0.001), DM (*p* = 0.026), ADF (*p* = 0.015), and NDF (*p* < 0.001) were lower in LIRG + PL than LIRG and HIRG. The ME and crude protein (CP) content of herbage, however, were similar among the farmlets in both milking years.

In 2017/18, the season had a significant effect on herbage content of OM (*p* = 0.039), CP (*p* = 0.004) and ME (*p* = 0.04), where herbage OM% was greater in spring than autumn, CP% was greater in autumn than spring and summer, and ME (MJ/kg DM) was greater in spring than summer. In 2018/19, the season had no effect on the chemical composition or ME content of herbage. There was no interaction effect (*p* > 0.05) on herbage chemical composition between season and farmlet.

### 3.2. Herbage Botanical Composition

**Figure 1:**
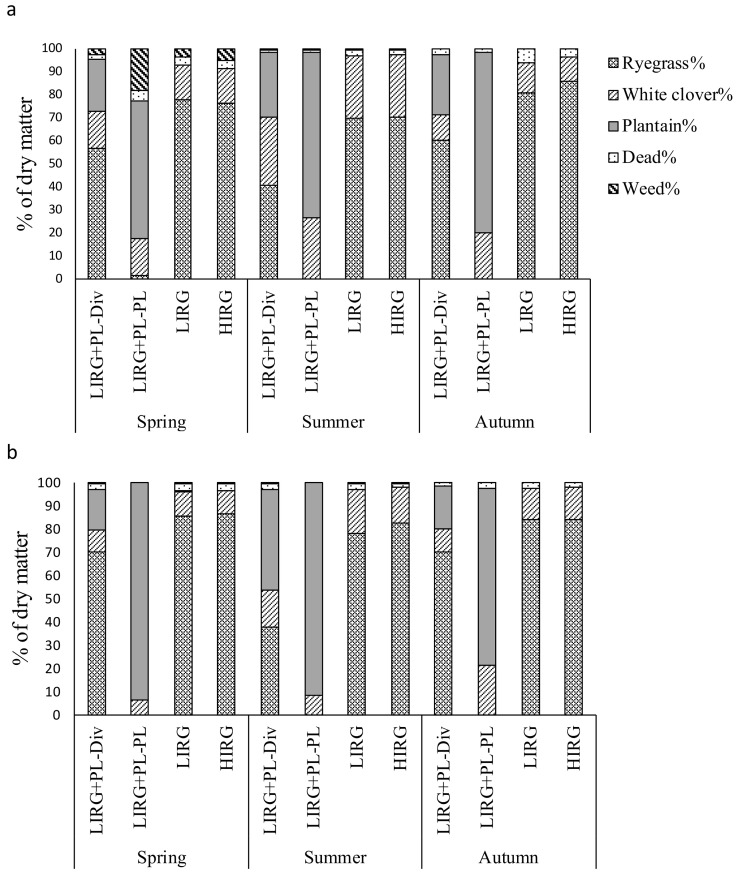
The botanical composition of herbage grazed by dairy cows in lower input ryegrass + plantain-based system (LIRG + PL; 60% of farm area on diverse mix of ryegrass, white clover, and plantain, and 40% of farm area on plantain–white clover mix, 3.5 cow/ha stocking rate, and 150 kg nitrogen/ha/year), lower input ryegrass-based system (LIRG; perennial ryegrass–white clover mix sward, 3.5 cow/ha, and 150 kg nitrogen/ha/year), and higher input ryegrass-based system (HIRG; perennial ryegrass–white clover mix sward, 5 cow/ha, and 300 kg nitrogen/ha/year) collected in spring, summer, and autumn in (**a**) 2017/18 and (**b**) 2018/19 milking years.

The mean botanical composition of herbage grazed by cows during spring, summer, and autumn in 2017/18 and 2018/19 milking years is presented in Figure 1. Average percentage of plantain was 69% (2017/18) and 84% (2018/19) in the plantain–white clover sward and 25% (2017/18) and 24% (2018/19) in the diverse sward grazed by cows in the LIRG + PL farmlet. The average percentage of white clover was similar (*p* > 0.05) across the swards within both milking years but was higher in 2017/18 (21%) than 2018/19 (13%). In both milking years, the average percentage of ryegrass did not differ between LIRG and HIRG (79%), which was greater (*p* < 0.001) than in the diverse sward of LIRG + PL (56%). The average percentages of weeds (1.7%) and dead material (2.3%) in the swards were low for all farmlets in both milking years.

In both milking years, the percentage of plantain in the diverse sward of LIRG + PL was affected by the season (*p* < 0.05), with a greater percentage of plantain in summer (32%) than spring (21%) and autumn (22%). The percentage of weeds in the plantain-dominant sward was greater (*p* < 0.05) in spring (18%) than summer (1%) and autumn (0%) in 2017/18, but similarly low across the seasons (<1%) in 2018/19.

### 3.3. Herbage Harvested and Supplement Fed

**Figure 2:**
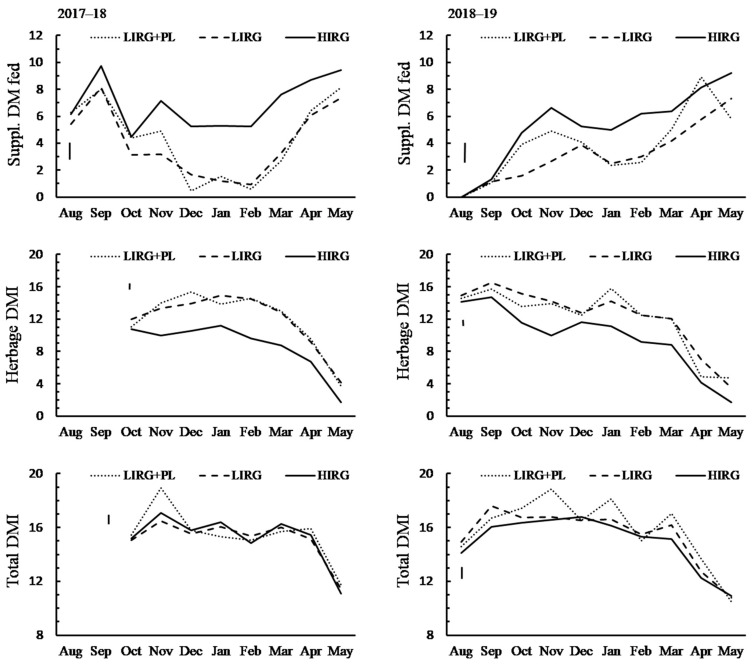
Estimated daily dry matter intake (DMI; kg/cow) of herbage (calculated based on individual cow energy output), supplement fed (mixture of maize silage and lucerne silage or grass silage), and total DMI (herbage + supplement; assuming supplement utilisation of 100%) of dairy cows grazing ryegrass and plantain-based swards in a lower input system (3.5 cow/ha stocking rate and 150 kg nitrogen/ha/year; LIRG + PL), perennial ryegrass–white clover mix sward in a lower input system (3.5 cow/ha and 150 kg nitrogen/ha/year; LIRG), or perennial ryegrass–white clover mix sward in a higher input system (5 cow/ha and 300 kg nitrogen/ha/year; HIRG) collected over two milking years, 2017/18 and 2018/19. Bars indicate LSD for farmlet effect within months.

The daily supplement fed and estimated daily herbage and total DMI during the lactation months are presented in Figure 2. There was a significant interaction effect (*p* < 0.001) between farmlet and months on DMI of cows among both milking years. In 2017/18, supplement fed to cows (kg DM/cow/day) was greater in HIRG than LIRG and LIRG + PL during the period from November to May and was lower for LIRG than LIRG + PL in October and November. Herbage DMI (kg/cow/day) was lower in HIRG than LIRG and LIRG + PL during the period from November to May and was lower in December but greater in October and January in LIRG compared to LIRG + PL. The total DMI (kg/cow/day) was greater in November and lower in January for LIRG + PL than LIRG and HIRG. In 2018/19, supplement fed (kg DM/cow/day) was greater in HIRG than LIRG from October to May, lower in LIRG + PL than HIRG in November, January, February, and May, and greater in LIRG + PL than LIRG in October, November, and April. During all lactation months except April, herbage DMI (kg/cow/day) was lower in HIRG than LIRG and LIRG + PL. Herbage DMI was greater in October and April but lower in January for LIRG than LIRG + PL. Total DMI (kg/cow/day) was lower in HIRG than LIRG and LIRG + PL in September and March and greater in LIRG + PL than LIRG farmlet in November, January, and April.

The grazing rotation intervals, compressed pre-grazing herbage height, and cumulative intake of herbage, supplement, total DM, and ME are presented in Table 3. Across both milking years, the average grazing rotation interval was longer in spring (49.2 days) than summer (21.3 days) and autumn (23.0 days). In the spring of both milking years, the compressed pre-grazing herbage height was greater (*p* < 0.001) for HIRG than LIRG + PL and LIRG and similar between LIRG + PL and LIRG. In autumn of 2018/19, the LIRG had greater (*p* < 0.001) compressed pre-grazing herbage height than LIRG + PL and HIRG.

A similar trend for cumulative herbage DMI and supplement fed was shown in both milking years. The cumulative herbage DMI t per cow was lower (*p* < 0.001) and t per ha was greater (*p* < 0.001) for HIRG than LIRG + PL and LIRG, and both were similar between LIRG + PL and LIRG. Cumulative supplement fed (t DM per cow and ha) was greatest for HIRG, lowest for LIRG, and intermediate for LIRG + PL (*p* < 0.001). Total DMI (kg per ha) was greater (*p* < 0.001) and total ME fed (MJ/cow) was lower (2018/17 *p* = 0.002; 2018/19 *p* < 0.001) for HIRG than LIRG + PL and LIRG, and both were similar between LIRG + PL and LIRG. The total DMI t per cow was similar among farmlets in 2017/18, but lower (*p* < 0.001) for HIRG than LIRG + PL and LIRG in 2018/19.

In 2017/18, the proportion of supplement fed in a cow’s diet (at DM bases) was greater (*p* < 0.001) for HIRG (45.1%) than LIRG + PL (26.2%) and LIRG (25.4%) and similar between LIRG + PL and LIRG. In 2018/19, the proportion of supplement fed in a cow’s diet was greatest (33.1%) in HIRG, lowest in LIRG (18.4%), and intermediate for LIRG + PL (23.1%) (*p* < 0.001).

### 3.4. Milk Production, LW, and BCS

The lactation length, annual milk yield, fat, protein, and MS production are shown in Table 4. In 2017/18, lactation length was longer (*p* = 0.012) for LIRG (280.6 day) than HIRG (276.0 day), with LIRG + PL (277.5 day) being intermediate. The annual milk yield, protein, fat, and MS production were lower in terms of kg per cow and greater in terms of kg per ha for HIRG than LIRG + PL and LIRG, and all were similar between LIRG + PL and LIRG. In 2018/19, lactation length was similar among the farmlets. The annual milk yield and protein production kg per cow were greatest for LIRG + PL, lowest for HIRG, and intermediate for LIRG (*p* < 0.001). The annual milk protein production kg per ha, however, was greater (*p* < 0.001) for HIRG than LIRG + PL and LIRG. The fat and MS production kg per cow were lower (*p* < 0.001) for HIRG than LIRG + PL and LIRG but were higher for HIRG in terms of kg per ha (*p* < 0.001).

The average LW and BCS values for cows across early, mid, and late stages of lactation in 2017/18 and 2018/19 milking years are presented in Table 4. In 2017/18, the average cow LW across the early lactation was greater (*p* = 0.01) in HIRG than LIRG + PL but similar between HIRG and LIRG and between LIRG and LIRG + PL. Farmlet did not affect the average cow LW during mid- and late lactation, or average cow BCS across all stages of lactation. In 2018/19, the average cow LW was greater in LIRG + PL than HIRG during early (*p* = 0.042), mid (*p* = 0.006), and late (*p* = 0.015) stage of lactation, but similar between LIRG + PL and LIRG and between LIRG and HIRG. The average cow BCS during mid-lactation was lower in HIRG than LIRG + PL and LIRG and similar between LIRG + PL and LIRG. Farmlet did not affect the average BCS of cows during early and late lactation.

### 3.5. Economics

Farm revenue, working expenses, and profit are presented in Table 5. The average farm revenue across both milking years was 31% and 32% greater per ha, 8% and 7% lower per cow, and similar per kg MS in HIRG compared to LIRG + PL and LIRG, respectively. However, the average farm working expenses were greater per ha (46% and 50%), per cow (2% and 5%), and per kg MS (13% and 15%) in HIRG than LIRG + PL and LIRG, respectively. The average farm profit was 11% and 9% greater per ha, but 22% and 23% lower per cow and 15% and 17% lower per kg MS in HIRG compared to LIRG + PL and LIRG, respectively. In comparison to the LIRG farmlet, the LIRG + PL farmlet showed a very similar per ha, per cow, and per kg MS farm revenue (less than 1% difference), with less than a 3% increase in farm working expenses and less than a 2.5% reduction in profitability.

### 3.6. Farm N Losses

Farm N surplus, nitrate leaching losses, and nitrous oxide emission are presented in Table 6. The average farm N surplus across both milking years was 36% greater for HIRG than LIRG and LIRG + PL. The average on-farm nitrate N leaching loss was 31% and 56% greater for HIRG than the LIRG and LIRG + PL, respectively, and 35% lower for the LIRG + PL than the LIRG. Average nitrous oxide emission was 29% and 34% greater for HIRG than LIRG and LIRG + PL, respectively, and 8% lower for LIRG + PL than LIRG.

## 4. Discussion

The objective of this study was to provide multiple-year and farm-scale evidence of the effects of integrating plantain and Italian ryegrass into a conventional New Zealand grazing dairy system on farm productivity, economic efficiency, and N losses. This is the first farm system study comparing the productivity, profitability, and nitrate leaching losses of a plantain plus Italian ryegrass-based dairy system with two common RGWC-based dairy systems differing in their levels of intensification (high and low input systems).

### 4.1. Farm Productivity and Profitability

The average herbage yield harvested by cows across the farmlets over both milking years in this study was 12 t DM/ha, which is lower than the average of 15.4 t DM/ha reported by Chapman, Dalley [33] in a study conducted at the same region. The Balmoral shallow stony soil at the site of current study has a lower water holding capacity compared to the deep Templeton silt loam soil at the site of Chapman, Dalley [33] study and, therefore, the ability to maintain soil moisture content for optimal herbage growth was lower in this study (New Zealand Soil Bureau, 1968). This lower yield was despite average annual rainfall (644 mm) being similar and applied irrigation (550 mm) being greater in this study than the study of Chapman, Dalley [33] (average annual rainfall = 655 mm; irrigation = 380 mm). At several irrigation intervals in Canterbury region, McBride [34] reported an average reduction of 30% in herbage DM yield per ha on the shallow soil (stone to the surface) compared to deep soil (no stone down to 600 mm). Therefore, the shallow stony composition of the soil at the site of this study has resulted in a relatively lower herbage DM yield per ha compared to the regional average.

The average annual herbage harvested per ha was similar between LIRG + PL (11.6 t DM/ha) and LIRG (11.7 t DM/ha) but increased with the increase in N fertiliser rate in HIRG (12.7 t DM/ha). This suggests that DM response to N fertiliser and overall growth potential of the plantain + Italian ryegrass swards used in LIRG + PL was similar to the conventional RGWC sward used in LIRG. Martin, Edwards [30] reported a similar DM yield response to N fertiliser between perennial ryegrass and plantain (19.7 and 19.3 kg DM/ha per 1 kg of N fertiliser, respectively). In addition, Woodward, Waugh [35] reported similar herbage DM yield between diverse sward containing plantain and chicory and a conventional RGWC sward. In contrast, Nobilly, Bryant [36] reported an average of 10% increase in the DM yield of a diverse sward containing ryegrass, lucerne, tall fescue, red clover, white clover, plantain, and chicory compared to the simple sward (ryegrass or tall fescue and white clover). Compared to this study, the high number of forage species in the sward used by Nobilly, Bryant [36] may have increased the diversity of functional traits, thus providing a chance for each species to fully utilise available resources (e.g., water, space) during different seasons (hot summer and cold winter) and resulting in an improved herbage yield (see also Pembleton, Tozer [37]). These results suggest that under the conditions of this study, the integration of plantain and Italian ryegrass into the dairy system can maintain farm herbage productivity at the same level as the conventional RGWC-based dairy system.

Herbage of similar quality, as reflected in the similar ME content of herbage among the farmlets, was fed to cows within the farmlets. This suggests that the high SR in HIRG did not result in a greater rate of herbage utilisation and herbage quality, compared to LIRG + PL and LIRG, as suggested by McCarthy, Delaby [38]. It is proposed that the low herbage yield per ha in this study resulted in the SR of 3.5 cows per ha being high enough to improve herbage utilisation and maximise herbage quality.

The diverse and plantain-dominant swards in the LIRG + PL farmlet were managed in a similar way to the conventional RGWC sward in LIRG. During both milking years, grazing rotation intervals, pre-grazing herbage height, and cumulative herbage intake per cow were similar between LIRG + PL and LIRG. However, quantity of supplement fed to cows was 4% and 30% greater for LIRG + PL than LIRG in 2017/18 and 2018/19, respectively. Considering the similar herbage quantity harvested per ha, the higher amount of supplement fed to cows in LIRG + PL compared to LIRG may reflect the greater plantain contribution in the cows’ diet during the hot months and the deficit during the cold months (autumn). However, the large increase in the amount of supplement fed to cows in LIRG + PL compared to LIRG in 2018/19 resulted in 6% and 5% increase in milk yield and milk protein kg per cow, respectively. Several component studies reported similar or improved milk production for grazing dairy cows fed plantain at different levels compared to those fed RGWC [6,10,11,19,31,39]. In this study, milk production and farm profit (<5% numerical difference) were similar between LIRG + PL and LIRG in 2017/18, and the increase in milk production and subsequent revenue for LIRG + PL compared to LIRG in 2018/19 offset the increase in the cost of purchased feed, resulting in a similar farm profit (<2% numerical difference). This result suggests that under the conditions of this study, the integration of plantain and Italian ryegrass into the dairy system could maintain farm productivity and profitability.

The average farmlet comparative stocking rate (CSR; kg LW per t of DM offered) [40] across the two milking years was 85, 86, and 92 kg LW per t DM for LIRG + PL, LIRG, and HIRG, respectively. The increase in CSR (i.e., increase in cow LW relative to the amount of feed offered per ha) in HIRG compared to LIRG + PL and LIRG reduced the MS production per cow by an average of 40 kg, due to less feed offered per cow. This corresponds to a 6.0 kg reduction in MS per one unit increase in CSR, which is comparable to the 4.5 kg MS per one unit of CSR reported by Macdonald, Penno [40] and Chapman, Dalley [33]. However, this reduction in milk production per cow in HIRG was compensated by the greater number of cows per ha, resulting in a greater average milk production per ha compared to LIRG + PL and LIRG. Subsequently, farm profitability, in NZD per ha, was on average 10% greater for the HIRG than LIRG + PL and LIRG. This is similar to the 9% increase in farm profit per ha reported by Hedley, Kolver [41] when a dairy system was intensified by increasing SR from 3.4 to 3.6 cow/ha and supplement offered from 1.6 to 2.6 kg DM per cow (60% increase), and the herbage harvested per ha was similar to this study (12 t DM/ha). In contrast, Chapman, Dalley [33] reported similar farm profit (NZD/ha) between lower (SR = 3.5 cow/ha and fertiliser rate of 150 kg N/ha) and higher (SR = 5.0 cow/ha and fertiliser rate of 300 kg N/ha) input dairy systems. Herbage is the cheapest source of feed for cows in the grazing dairy system, but in this study, the low herbage yield resulted in a high additional cost for purchased feed in all farmlets (an average of NZD 27,900, NZD 24,500, and NZD 58,000 for LIRG + PL, LIRG and HIRG, respectively). The cost of purchased supplement fed to cows during the lactation period (from calving to dry-off) was not determined in the study of Chapman, Dalley [33], but cows in the low input system required 85% less supplementary DM and produced 24% less MS kg per ha than those in the high input system. In our study, however, cows in the lower input system (LIRG + PL and LIRG) were fed an average of 38% less supplement DM and produced 23% less MS kg per ha than those in the higher input system (HIRG). Therefore, the high feed cost for cows in the lower input systems in this study may have contributed to a lower profitability per ha for LIRG and LIRG + PL compared to HIRG. Clearly, the economic benefits of intensified dairy systems will depend on the additional feed cost per ha and the milk production response to that additional feed.

### 4.2. Farm N Losses

Deintensification of pastoral grazing dairy systems has been linked to a decrease in farm N surpluses and subsequently lower nitrate leaching losses into water resources [33]. In this study, two different input intensities of the same sward mix (LIRG and HIRG) showed that deintensification under the LIRG pasture system decreased the annual estimated farm N surplus by 36% and subsequently decreased the nitrate leaching loss by 32%. Similarly, Chapman, Dalley [33] reported a 46% decrease in annual N surplus and a 25% decrease in estimated nitrate leaching losses by decreasing the dairy system SR from 5.0 down to 3.5 cow/ha, per cow imported feed by 85%, and applied N fertiliser rate from 300 to 150 to kg N/ha. In addition, the estimated nitrous oxide emission in this study was decreased by 29% when the dairy system was deintensified by decreasing SR, N fertiliser rate, and per cow supplement (LIRG vs. HIRG). The small decrease in farm profitability achieved by deintensifying a dairy system can make a significant reduction in environmental impact.

The objective of incorporating plantain into the dairy system was to further reduce the farm nitrate leaching losses. This is considered to occur through the reduced urinary N concentration and subsequently a lower N load per urine patch of cows grazing swards containing plantain compared to those grazing RGWC [3,8,10]. The mechanism by which plantain reduces urinary N concentration is not fully known but has been attributed to water diuresis caused by low DM content in plantain, combined with the diuretic effect of the high content of minerals and secondary compounds, both leading to a large urine volume [10,19,20,42]. Recent research has shown that a minimum of 30% of plantain (DM basis) in the diet of dairy cows is required to elicit such an effect and reduce urinary N concentration [19]. This is in agreement with previous studies that showed no difference in urinary N concentration from cows which grazed swards containing <20% plantain [35,43]. In addition, other studies have shown a reduction in urinary N concentration when plantain was included at >40% in the diet of dairy cows [10,11,20,21]. In this study, the average proportion of plantain in the diet of cows (estimated as herbage DMI multiplied by plantain % in the sward divided by total DMI) was 33% in 2017/18 and 41% in 2018/19. However, the daily proportion of plantain in the diet ranged from 4% (when cows grazed on the diverse sward and the proportion of supplement in the diet was high) to 94% (when cows grazed on the plantain dominant sward and the proportion of supplement in the diet was low). This suggests that in farming systems like ours, a plantain-induced reduction in urinary N concentration would not occur every day and so not be detected by urine spot samples used to estimate long-term trends in N excretion. In addition, the benefit of plantain in reducing nitrate leaching losses is achieved through altering the urination behaviour of cows (i.e., a higher frequency of low N concentration urination events) compared to cows fed RGWC [10,44]. Such an effect should be directly measured at paddock scale rather than in individual animals and over a long-term farm system study rather than short-term lysimeter study.

The effect of plantain on nitrate leaching losses was not directly measured in this study but was estimated using a modelling tool (Overseer^®^) that is sensitive to the effect of plantain on urinary N excretion by cows. At similar farm annual N surplus, Overseer^®^ showed that the integration of plantain into the RGWC dairy system reduced the estimated annual nitrate N leaching losses by 35% (i.e., 17.5 kg N/ha for LIRG + PL and 27 kg N/ha for LIRG). This is much higher than the simulated 7% reduction in nitrate N leaching reported by Bryant, Snow [5] for cows fed diuretic components of lucerne and plantain compared to those fed RGWC, when extrapolated to paddock scale. Perhaps this discrepancy in results confirms the need of further studies and methods that directly measure the benefit of plantain, and Italian ryegrass, in reducing N leaching at farm scale.

In addition to perennial ryegrass, white clover, and plantain, the diverse sward in the LIRG + PL contained Italian ryegrass. The major reason for the inclusion of Italian ryegrass in the sward of LIRG + PL was to increase the growth rate during the cool season [14,45]. Italian ryegrass has greater winter growth, compared to herbage species commonly used in the grazing system, allowing more N uptake [13,35,45]. In a lysimeter study, Maxwell, McLenaghen [13] reported greater annual DM yield and N uptake with Italian ryegrass, mainly due to an increase in growth rate in winter and early spring, and subsequent 33–46% reduction in the annual nitrate leaching losses under Italian ryegrass compared to perennial ryegrass. The model used in this study does not take into account the effect of Italian ryegrass on N leaching; however, herbage harvested (kg DM ha) and pre-grazing herbage height during spring were similar between LIRG + PL and LIRG, suggesting that the overall herbage growth rate was unlikely to have been influenced by the inclusion of Italian ryegrass in the sward. The proportion of Italian ryegrass in the sward was not quantified in this study but perhaps was not high enough to elicit such effect. Another possibility is that the growth advantage of Italian ryegrass during the cool season was offset by the slow growth of plantain, resulting in similar overall sward growth to that of RGWC. More research on the incorporation of Italian ryegrass into the dairy system is required, and the expected environmental benefits, N uptake and nitrate leaching losses, need to be directly measured at farm level.

## 5. Conclusions

Regardless of herbage type, under the conditions of this study, deintensifying the grazing dairy system by decreasing the SR, fertiliser rate, and level of imported feed decreased estimated nitrate leaching losses by 32%, whilst farm profitability decreased by 10%. Under similar management and farming input systems, results of this study also showed that the integration of plantain and Italian ryegrass into the RGWC-based dairy system reduces estimated nitrate leaching losses further while maintaining farm productivity and profitability. Therefore, an Italian ryegrass plus plantain-based dairy pasture system could be considered as an alternative option to the conventional RGWC-based system to help farmers to meet regulations to reduce their farm environmental footprint without reducing farm productivity and profitability. However, the environmental benefits of plantain and Italian ryegrass estimated in this study require further confirmation through direct measurements at farm scale.

## Figures and Tables

**Table 1:** Dry matter (DM) %, organic matter (OM), crude protein (CP), acid detergent fibre (ADF) and neutral detergent fibre (NDF) % in DM, and metabolisable energy (ME; MJ/kg DM) of supplement fed to cows during 2017/18 and 2018/19 milking year. Mean ± SD.

Milking Year	Type	OM%	DM%	CP%	ADF%	NDF%	ME
2017/18	Maize silage	96.3 ± 2.46	39.2 ± 3.44	7.1 ± 1.56	23.1 ± 4.00	43.5 ± 8.17	11.1 ± 0.56
	Lucerne Silage	90.7 ± 1.93	51.4 ± 9.63	18.5 ± 4.00	33.9 ± 6.10	43.3 ± 3.82	9.4 ± 0.50
2018/19	Maize silage	96.2 ± 0.99	35.4 ± 2.48	7.4 ± 0.27	26.8 ± 1.65	46.6 ± 2.81	10.1 ± 0.33
	Grass silage	92.0 ± 1.86	49.1 ± 14.87	13.2 ± 3.58	35.3 ± 4.09	56.4 ± 7.39	9.4 ± 1.26
	Lucerne Silage	89.8 ± 1.58	44.8 ± 12.96	22.1 ± 1.75	32.3 ± 2.56	39.8 ± 2.59	9.8 ± 0.62

**Table 2:** Dry matter (DM) %, organic matter (OM), crude protein (CP), acid detergent fibre (ADF) and neutral detergent fibre (NDF) % in DM, and metabolisable energy (MJ/kg DM) of herbage grazed by cows during spring, summer, and autumn in 2017/18 and 2018/19 milking year.

Milking Year	Farmlet ^1^	Season	OM%	DM%	CP%	ADF%	NDF%	ME
2017/18	LIRG + PL	Spring	89.7	16.9	19.6	22.2	35.6	12.3
		Summer	87.5	16.5	20.8	24.0	34.0	11.7
		Autumn	86.9	12.3	25.3	21.4	32.7	11.8
		Whole year	88.0	15.2	21.9	22.5	34.1	11.9
	LIRG	Spring	91.2	19.2	18.2	23.3	41.6	12.4
		Summer	90.3	20.4	21.4	24.8	42.5	11.7
		Autumn	89.5	15.2	26.0	24.2	42.9	12.0
		Whole year	90.3	18.3	21.9	24.1	42.3	12.1
	HIRG	Spring	90.7	19.7	18.3	23.3	41.9	12.2
		Summer	90.0	19.6	22.0	24.9	43.1	11.6
		Autumn	89.8	15.6	26.3	24.7	44.6	11.8
		Whole year	90.2	18.3	22.2	24.3	43.2	11.8
		*LSD farmlet*	1.1	1.4	1.2	1.0	3.1	0.2
		*LSD season*	1.4	4.4	2.9	2.6	4.3	0.5
		*P farmlet*	<0.001	<0.001	0.558	0.008	<0.001	0.100
		*P season*	0.039	0.082	0.004	0.318	0.965	0.040
2018/19	LIRG + PL	Spring	90.4	18.3	21.4	20.9	37.3	12.6
		Summer	89.3	15.3	19.3	22.3	30.5	11.9
		Autumn	89.0	12.0	25.3	21.3	31.6	12.2
		Whole year	89.5	15.2	22.0	21.5	33.1	12.2
	LIRG	Spring	90.4	18.8	22.6	22.3	40.7	12.5
		Summer	90.7	16.3	22.3	23.9	41.1	12.0
		Autumn	91.0	14.7	24.6	23.9	42.2	12.2
		Whole year	90.7	16.6	23.2	23.4	41.3	12.2
	HIRG	Spring	90.7	19.8	21.3	22.1	40.4	12.6
		Summer	90.2	16.5	23.8	23.5	40.2	11.9
		Autumn	90.9	15.1	25.8	23.3	41.4	12.2
		Whole year	90.6	17.2	23.6	23.0	40.6	12.2
		*LSD farmlet*	0.6	1.4	1.6	1.3	4.2	0.3
		*LSD season*	1.7	4.5	6.0	3.7	8.8	0.8
		*P farmlet*	<0.001	0.026	0.141	0.015	<0.001	0.991
		*P season*	0.798	0.059	0.335	0.543	0.784	0.141

^1^ LIRG + PL = lower input system on ryegrass + plantain (perennial ryegrass, Italian ryegrass, white clover, and plantain diverse mix, and plantain–white clover mix sward, 3.5 cow/ha, and 150 kg nitrogen/ha/year); LIRG = lower input system on ryegrass (perennial ryegrass–white clover mix sward, 3.5 cow/ha, and 150 kg nitrogen/ha/year); HIRG = higher input system on ryegrass (perennial ryegrass–white clover mix sward, 5.0 cow/ha, and 300 kg nitrogen/ha/year).

**Table 3:** Grazing rotation intervals (mean ± SD), compressed pre-grazing herbage height, herbage dry matter intake (DMI), supplement dry matter (DM) fed and % in diet, total DMI, and total metabolisable energy (ME) fed to dairy cows grazing ryegrass and plantain-based swards in a lower input system (LIRG + PL; 3.5 cow/ha stocking rate and herbage received 150 kg nitrogen/ha/year), perennial ryegrass–white clover mix sward in a lower input system (LIRG; 3.5 cow/ha and herbage received 150 kg nitrogen/ha/year), or perennial ryegrass–white clover mix sward in a higher input system (HIRG; 5 cow/ha and herbage received 300 kg nitrogen/ha/year) during the lactation period (from August to May) in 2017/18 and 2018/19 milking years.

Item	2017/18					2018/19				
	LIRG + PL	LIRG	HIRG	LSD	*p* Value	LIRG + PL	LIRG	HIRG	LSD	*p* Value
Grazing rotation (d)										
Spring	56.4 ± 32.1	56.4 ± 32.1	56.7 ± 31.8			43.7 ± 26.8	42.4 ± 27.0	42.3 ± 26.7		
Summer	21.6 ± 0.8	20.0 ± 0.0	22.0 ± 0.0			21.9 ± 2.5	21.9 ± 2.5	20.0 ± 0.0		
Autumn	22.9 ± 2.6	22.9 ± 2.6	22.0 ± 0.0			25.4 ± 1.4	25.4 ± 1.4	20.4 ± 1.4		
Pre-grazing herbage height (cm)										
Spring	6.76	6.84	7.46	0.32	<0.001	8.25	8.54	8.99	0.32	<0.001
Summer	7.64	7.64	7.61	0.45	0.994	7.87	7.83	7.82	0.23	0.867
Autumn	6.70	6.68	6.71	0.28	0.977	6.58	7.04	6.80	0.24	<0.001
Herbage DMI										
t/cow	3.30	3.33	2.45	0.137	<0.001	3.27	3.37	2.64	0.151	<0.001
t/ha	11.54	11.65	12.24	0.580	0.019	11.71	11.8	13.21	0.638	<0.001
Supplement fed (DM)										
t/cow	1.12	1.07	1.88	0.017	<0.001	0.98	0.75	1.29	0.031	<0.001
t/ha	3.91	3.75	9.40	0.077	<0.001	3.42	2.61	6.50	0.136	<0.001
% in diet	26.2	25.4	45.1	1.13	<0.001	23.1	18.4	33.1	0.95	<0.001
Total DMI ^1^										
t/cow	4.29	4.26	4.22	0.137	0.535	4.26	4.11	3.94	0.158	<0.001
t/ha	15.02	14.89	21.10	0.590	<0.001	14.37	14.90	19.71	0.651	<0.001
Total ME fed (MJ/cow)	49,309	49,400	47,122	1610.2	0.002	49,678	48,402	44,939	1931.0	<0.001

^1^ Total DMI = herbage intake + supplement fed, assuming supplement utilisation of 100%.

**Table 4:** Lactation length, annual milk yield, milk solids (sum of fat and protein), fat, and protein production, and live weight (LW) and body condition score (BCS; 1–10 scale) values averaged within early (≤100 days in milk), mid (>100 and ≤200 days in milk), and late (>200 days in milk) stage of lactation of dairy cows grazing ryegrass and plantain-based swards in a lower input system (3.5 cow/ha stocking rate and herbage received 150 kg nitrogen/ha/year; LIRG + PL), perennial ryegrass–white clover mix sward in a lower input system (3.5 cow/ha and herbage received 150 kg nitrogen/ha/year; LIRG), or perennial ryegrass–white clover mix sward in a higher input system (5 cow/ha and herbage received 300 kg nitrogen/ha/year; HIRG) in 2017/18 and 2018/19 milking years.

Item	2017/18					2018/19				
	LIRG + PL	LIRG	HIRG	LSD	*p* Value	LIRG + PL	LIRG	HIRG	LSD	*p* Value
Lactation length (d)	277.5	280.6	276.0	3.25	0.012	257.8	256.5	255.1	7.40	0.723
Annual milk production										
Yield (kg/cow)	4823	4752	4571	259.8	0.096	5184	4909	4505	227.5	<0.001
Protein										
kg/cow	196.1	196.0	180.9	8.34	<0.001	204.4	195.1	177.3	8.87	<0.001
kg/ha	686.4	685.9	904.3	35.79	<0.001	715.5	682.9	886.5	36.93	<0.001
Fat										
kg/cow	254.0	259.7	244.1	13.55	0.042	281.8	273.7	249.1	14.30	<0.001
kg/ha	888.4	909.1	1220.3	57.46	<0.001	986.2	957.8	1245.3	58.94	<0.001
Milk solids										
kg/cow	449.9	455.7	424.9	21.22	0.004	486.3	471.1	426.4	22.45	<0.001
kg/ha	1575	1595	2125	90.4	<0.001	1702	1649	2132	93.0	<0.001
LW (kg)										
Early lactation	421.4	432.4	443.0	15.1	0.010	458.1	449.3	440.9	13.6	0.042
Mid lactation	455.9	458.1	464.5	13.9	0.379	474.0	461.7	452.0	14.5	0.006
Late lactation	478.7	480.8	481.5	14.3	0.913	483.9	479.7	465.8	14.2	0.015
BCS (units)										
Early lactation	4.19	4.15	4.18	0.13	0.767	4.43	4.43	4.39	0.13	0.782
Mid lactation	4.19	4.15	4.21	0.11	0.467	4.37	4.27	4.26	0.10	0.048
Late lactation	4.20	4.22	4.20	0.10	0.921	4.27	4.30	4.28	0.09	0.810

**Table 5:** Farmax model economic output (in New Zealand dollar) for lower input ryegrass and plantain-based (LIRG + PL; perennial ryegrass, Italian ryegrass, white clover, and plantain diverse mix, and plantain–white clover mix sward, 3.5 cow/ha and herbage received 150 kg nitrogen/ha), lower input ryegrass-based (LIRG; perennial ryegrass–white clover mix sward, 3.5 cow/ha and herbage received 150 kg nitrogen/ha) and higher input ryegrass-based (HIRG; perennial ryegrass–white clover mix sward, 5 cow/ha and herbage received 300 kg nitrogen/ha) irrigated diary systems. The model financial outputs are based on actual production input data collected from each farm system over two lactation years, 2017/18 and 2018/19, and at milk price of NZD 6.5 per kg of milk solids (sum fat + protein).

Lactation Seasons	Farmlet	Farm Revenue			Farm Working Expenses			Farm Profit (Before Tax)		
		NZD/ha	NZD/cow	NZD/kg Milk Solid	NZD/ha	NZD/cow	NZD/kg Milk Solid	NZD/ha	NZD/cow	NZD/kg Milk Solid
2017/18	LIRG + PL	10,390	2960	6.58	6230	1780	3.95	3740	1060	2.37
	LIRG	10,540	3000	6.58	6150	1750	3.84	3950	1130	2.47
	HIRG	14,090	2810	6.64	9470	1890	4.46	4200	837	1.98
2018/19	LIRG + PL	11,140	3170	6.53	6310	1800	3.70	4400	1250	2.58
	LIRG	10,800	3080	6.52	6060	1730	3.66	4320	1230	2.61
	HIRG	14,160	2820	6.61	8900	1770	4.16	4830	962	2.25

**Table 6:** Estimated nitrogen (N) surplus, nitrate leaching loss and nitrous oxide (N_2_O) N loss (kg N/ha per year), and nitrous oxide emission (equivalent to CO_2_ tonne per year) for lower input ryegrass + plantain-based (LIRG + PL; perennial ryegrass, Italian ryegrass, white clover, and plantain diverse mix, and plantain–white clover mix swards, 3.5 cow/ha and herbage received 150 kg nitrogen/ha), lower input ryegrass-based (LIRG; perennial ryegrass–white clover mix sward, 3.5 cow/ha and herbage received 150 kg nitrogen/ha), and higher input ryegrass-based (HIRG; perennial ryegrass ryegrass–white clover mix sward, 5 cow/ha and herbage received 300 kg nitrogen/ha) irrigated diary systems over two milking years, 2017/18 and 2018/19. Values estimated using Overseer^®^ (Version 6.3.4).

Item		2017/18			2018/19	
	LIRG + PL	LIRG	HIRG	LIRG + PL	LIRG	HIRG
Nitrogen surplus	230.0	235.0	376.0	242.0	239.0	368.0
Nitrate N leaching	17.0	26.0	39.0	18.0	28.0	40.0
N_2_O N loss	5.8	6.3	8.9	6.2	6.7	9.3
N_2_O emission ^1^	60.0	65.0	92.0	64.0	69.0	96.0

^1^ Nitrous oxide global warming potential = 290 times that of CO_2_ for a 100-year timescale.
